# Beta-lactamase antimicrobial resistance in *Klebsiella* and *Enterobacter* species isolated from healthy and diarrheic dogs in Andhra Pradesh, India

**DOI:** 10.14202/vetworld.2017.950-954

**Published:** 2017-08-20

**Authors:** N. Mohammad Sharif, B. Sreedevi, R. K. Chaitanya, D. Sreenivasulu

**Affiliations:** Department of Veterinary Microbiology, College of Veterinary Science, Sri Venkateswara Veterinary University, Tirupati, Andhra Pradesh, India

**Keywords:** beta-lactamase resistance, dogs, *Enterobacter*, extended-spectrum beta-lactamase, *Klebsiella*

## Abstract

**Aim:**

The aim of this study was to characterize beta-lactamase antimicrobial resistance in *Klebsiella* and *Enterobacter* species isolated from healthy and diarrheic dogs in Andhra Pradesh.

**Materials and Methods:**

A total of 136 rectal swabs were collected from healthy (92) and diarrheic (44) dogs, bacteriological cultured for *Klebsiella* and *Enterobacter* growth and screened for beta-lactamase antimicrobial resistance phenotypically by disc diffusion method and genotypically by polymerase chain reaction targeting *bla*_TEM_, *bla*_SHV_, *bla*_OXA_, *bla*_CTX-M_ Group 1, 2, *bla*_AmpC_, *bla*_ACC_, and *bla*_MOX_ genes.

**Results:**

A total of 33 *Klebsiella* and 29 *Enterobacter* isolates were recovered. Phenotypic beta-lactamase resistance was detected in 66.6% and 25% of *Klebsiella* and *Enterobacter* isolates, respectively, from healthy dogs and 66.6% and 60% of *Klebsiella* and *Enterobacter* isolates, respectively, from diarrheic dogs. Overall, incidence of extended-spectrum beta-lactamase (ESBL) phenotype was found to be 21.2% (7/33) in *Klebsiella* isolates, whereas none of the *Enterobacter* isolates exhibited ESBL phenotype. Predominant beta-lactamase genes detected in *Klebsiella* species include *bla*_SHV_ (84.8%), followed by *bla*_TEM_ (33.3%), *bla*_CTX-M_ Group 1 (15.1%), and *bla*_OXA_ (6.1%) gene. Predominant beta-lactamase genes detected in *Enterobacter* species include *bla*_SHV_ (48.2%), followed by *bla*_TEM_ (24.1%), *bla*_AmpC_ (13.7%), and *bla*_OXA_ (10.3%) gene.

**Conclusion:**

The present study highlighted alarming beta-lactamase resistance in *Klebsiella* and *Enterobacter* species of canine origin in India with due emphasis as indicators of antimicrobial resistance.

## Introduction

Emergence of multidrug resistance among *Enterobacteriaceae* members isolated from companion animals has increased substantially over the past 20 years [1]. One resistance mechanism that is of particular concern is that mediated by a family of bacterial enzymes called beta-lactamases that confer resistance to beta-lactam antibiotics [2]. Beta-lactamase antimicrobial resistance typically develops as a consequence of selective pressure exerted by misuse of cephalosporins [2].

Gut microbiota acts as an ideal reservoir of antimicrobial resistance genes [1]. Overuse and misuse of antimicrobials disrupt normal gut microbiota and select resistant bacteria, leading to enrichment of antibiotic resistant populations inside gut and emergence of so-called “superbugs” [1]. Extended-spectrum beta-lactamases (ESBLs) are variants of beta-lactamases that confer resistance to the third-generation cephalosporins such as cefotaxime, ceftazidime, and ceftriaxone as well as to monobactams such as aztreonam [2]. Beta-lactamase production is mediated by beta-lactamase (*bla*) genes carried on a plasmid or on the chromosome [2]. Over the past few years, production of beta-lactamases among Gram-negative organisms has increased drastically, especially among the members of *Enterobacteriaceae* [1,3,4]. Investigating the level of resistance among commensal *Enterobacteriaceae* was considered as a good indicator of the prevalence of antimicrobial resistance [5].

Although there are reports of phenotypic and genotypic detection of beta-lactamase antimicrobial resistance in farm animals and poultry from India, only a few studies were undertaken in dogs. Keeping in view the above, the present study was conducted with an objective of detection of beta-lactamase antimicrobial resistance in *Klebsiella* and *Enterobacter* species isolated from healthy and diarrheic dogs.

## Materials and Methods

### Ethical approval

Ethical approval was not necessary for this study. However, samples were collected as per standard collection procedure without harming or giving stress to the animals.

### Reference strains

Beta-lactamase-positive strain of *Klebsiella pneumoniae* (American Type Culture Collection [ATCC] 700603) and beta-lactamase-negative strain of *E. coli* (ATCC 25922) were used as controls in this study.

### Collection and processing of samples

A total of 136 rectal swab samples were collected from healthy (n=92) and diarrheic (n=44) dogs. Isolation and identification of *Klebsiella* and *Enterobacter* species were carried out by conventional cultural methods and biochemical tests [6]. Whole-cell DNA was extracted by boiling and snap chilling method [7]. The absorbance of the DNA at wavelengths 260 nm and 280 nm was measured using Nanodrop (Thermo Scientific, USA).

### Phenotypic screening for beta-lactamase resistance

*Klebsiella* and *Enterobacter* isolates were subjected to antibiotic sensitivity testing by disc diffusion method on Mueller-Hinton agar [8]. Inhibition zone diameters were interpreted according to the Clinical and Laboratory Standards Institute (CLSI) guidelines [9,10]. CLSI recommends two-step procedure for the phenotypic detection of ESBL production, which includes an initial “screening test” to detect resistance against one or more indicator substrates followed by “confirmatory test” using one or more of the indicator substrates in combination with a beta-lactamase inhibitor, looking for synergy effects. Isolates were screened for resistance against four indicator antimicrobial agents: Cefotaxime (CTX, 30 µg), ceftazidime (CAZ, 30 µg), ceftriaxone (CTR, 30 µg), and aztreonam (AT, 30 µg). Resistance to at least one of the indicator antibiotics was considered as “positive” screening test [9,10].

### Phenotypic confirmation of ESBL production

Screening test positive isolates were subjected to “confirmatory test” by combination disc method. Three pairs of discs (i.e., with and without beta-lactamase inhibitor) were placed: Ceftazidime (CAZ, 30 µg), ceftazidime plus clavulanic acid (CAC, 30/10 µg), cefotaxime (CTX, 30 µg), cefotaxime plus clavulanic acid (CEC, 30/10 µg) and ceftriaxone (CTR, 30 µg), and ceftriaxone plus sulbactam (CIS, 30/10 µg). An isolate was confirmed for ESBL production when the inhibition zone diameter around combination discs was ≥5 mm (synergy effect) when compared to discs containing respective cephalosporin alone [9,10].

### Molecular detection of beta-lactamase (*bla*) genes

Three multiplex polymerase chain reaction (PCR) assays [11] and a single uniplex PCR [12] were standardized for the detection of beta-lactamase genes. The use of positive (positive DNA) and negative (nuclease free water) controls was adhered to in all the PCR assays. Oligonucleotide primers (M/s. Eurofins Genomics India Pvt. Ltd., Bengaluru, India) used, and their respective amplicon sizes were given in Table 1. All the other reagents used in the PCR assays were of Genei™, Bengaluru, India.

**Table-1:** Oligonucleotide primers used for the detection of betalactamase genes.

Primer	Primer sequence (5’-3’)	Amplicon size
Multiplex PCR-I (detection of beta-lactamase genes - TEM, SHV, and OXA)		
*bla_TEM_* gene	F: CATTTCCGTGTCGCCCTTATTC	800 bp
	R: CGTTCATCCATAGTTGCCTGAC	
*bla_SHV_* gene	F: AGCCGCTTGAGCAAATTAAAC	713 bp
	R: ATCCCGCAGATAAATCACCAC	
*bla_OXA_* gene	F: GGCACCAGATTCAACTTTCAAG	564 bp
	R: GACCCCAAGTTTCCTGTAAGTG	
Multiplex PCR-II (detection of beta-lactamase genes - CTX-M Group 1 and 2)		
*bla_CTX-M_* Group 1	F: TTAGGAAATGTGCCGCTGTA	688 bp
	R: CGATATCGTTGGTGGTACCAT	
*bla_CTX-M_* Group 2	F: CGTTAACGGCACGATGAC	404 bp
	R: CGATATCGTTGGTGGTACCAT	
Multiplex PCR-III (detection of beta-lactamase genes - ACC and MOX)		
*bla_ACC_*	F: CACCTCCAGCGACTTGTTAC	346 bp
	R: GTTAGCCAGCATCACGATCC	
*bla_MOX_*	F: GCAACAACGACAATCCATCCT	895 bp
	R: GGGATAGGCGTAACTCTCCCAA	
Uniplex PCR (detection of bla*_AmpC_* gene)		
*bla_AmpC_*	F: CCCCGCTTATAGAGCAACAA	631 bp
	R: TCAATGGTCGACTTCACACC	

PCR=Polymerase chain reaction

### Multiplex PCR-I for the detection of *bla_TEM_*, *bla_SHV_*, and *bla_OXA_* genes

Reaction mixture was optimized in 25 µl volume containing 2 µl of DNA template prepared from each isolate; *Taq* buffer (×10) - 3 µl; deoxynucleotide triphosphates (dNTPs) mix (10 mM) - 1 µl; MgCl_2_ (25 mM) - 1.5 µl; three forward primers (10 pmol/µl) - each 0.5 µl; three reverse primers (10 pmol/µl) - each 0.5 µl; *Taq* DNA polymerase (1 U/µl) - 1 µl; and nuclease free water - 13.5 µl.

### Multiplex PCR-II for the detection of *bla_CTX-M_* Group 1 and 2 genes

Reaction mixture was optimized in 25 µl volume containing 1.5 µl of DNA template prepared from each isolate; *Taq* buffer (×10) - 2.75 µl; dNTP mix (10 mM) - 0.5 µl; MgCl_2_ (25 mM) - 1 µl; two forward primers (10 pmol/µl) - each 0.75 µl; two reverse primers (10 pmol/µl) - each 0.75 µl; *Taq* DNA polymerase (1 U/µl) - 1 µl; and nuclease free water - 15.25 µl.

### Multiplex PCR-III for the detection of *bla_ACC_* and *bla_MOX_* genes

Reaction mixture was optimized in 25 µl volume containing 2 µl of DNA template prepared from each isolate; *Taq* buffer (×10) - 2.75 µl; dNTP mix (10 mM) - 1 µl; MgCl_2_ (25 mM) - 1.5 µl; two forward primers (10 pmol/µl) - each 0.6 µl; two reverse primers (10 pmol/µl) - each 0.6 µl; *Taq* DNA polymerase (1 U/µl) - 1 µl; and nuclease free water - 14.35 µl.

All the three multiplex PCR assays were carried out in Kyratec thermal cycler (Australia) under following standardized cycling conditions - initial denaturation at 94°C for 10 min, 30 cycles of denaturation at 94°C for 40 s, annealing at 60°C for 40 s, elongation at 72°C for 1 min, final elongation at 72°C for 7 min, and hold at 4°C.

### Uniplex PCR assay for the detection of *bla_AmpC_* gene

The PCR was optimized in 25 µl reaction mixture (containing 1 µl of DNA template prepared from each isolate; *Taq* buffer [×10] - 2.5 µl; dNTP mix [10 mM] - 0.5 µl; MgCl_2_ [25 mM] - 1.5 µl; forward primer [10 pmol/µl] - 1 µl; reverse primer [10 pmol/µl] - 1 µl; *Taq* DNA polymerase [1 U/µl] - 1 µl; and nuclease free water - 16.5 µl) under the following standardized cycling conditions: Initial denaturation of 94°C for 5 min, followed by 30 cycles of denaturation at 94°C for 30 s, annealing at 58°C for 30 s, and extension at 72°C for 30 s. Final extension was done at 72°C for 10 min.

## Results and Discussion

### Isolation and identification

On analysis of 136 rectal swab samples, a total of 33 (24.2%) *Klebsiella* and 29 (21.3%) *Enterobacter* isolates were recovered. From healthy dogs (92), 24 (26%) *Klebsiella* and *Enterobacter* isolates each were recovered. From diarrheic dogs (44), 9 (20.4%) *Klebsiella* and 5 (11.3%) *Enterobacter* isolates were recovered. In a study from Wisconsin (USA), *Klebsiella* and *Enterobacter* species were isolated from 57.1% and 42.8% of healthy dog fecal samples examined, respectively [13]. In a study from Chhattisgarh (India), *Klebsiella* species were isolated from 12% and 35% of healthy and diarrheic dog fecal samples, respectively [14]. Observed variation in the proportion of isolation across studies might be due to various variables such as genetic determinants, diet, environment, and age.

### Detection of beta-lactamase resistance

An overall incidence of 66.6% (22/33) and 31% (9/29) beta-lactamase resistance was detected in *Klebsiella* and *Enterobacter* species, respectively. Healthy and diarrheic dogs were isolated, respectively, 16 (66.6%)/6 (66.6%) *Klebsiella* and 6 (25.0%)/3 (60.0%) *Enterobacter* isolates. All these isolates were found to be resistant to one or more of beta-lactam antibiotics indicators and were designated as “suspect ESBL producers.” In a study from the Netherlands, 45% incidence of beta-lactamase resistance was reported in *Enterobacteriaceae* isolated from pet dogs [15]. Frequency of beta-lactam resistance detected in *Klebsiella* and *Enterobacter* isolates recovered in the present study was given in Table 2. Overall, the incidence of resistance to cefotaxime, ceftriaxone, ceftazidime, and aztreonam was found to be 42.4% (14/33), 36.3% (12/33), 33.3% (11/33), and 9.1% (3/33), respectively, in *Klebsiella* species. A study from France reported resistance to cefotaxime, ceftazidime, and aztreonam in all the 18 *Klebsiella* isolates recovered from hospitalized dogs [16]. Overall, the incidence of resistance to cefotaxime, ceftriaxone, and ceftazidime was found to be 24.1% (7/29), 17.2% (5/29), and 13.7% (4/29), respectively, in *Enterobacter* species. Aztreonam resistance was not detected in *Enterobacter* isolates recovered in this study. A study from Australia reported complete resistance to cefotaxime, ceftazidime, and aztreonam in all the 10 *Enterobacter* isolates recovered from hospitalized dogs [17].

**Table-2:** Frequency of beta-lactam resistance detected in *Klebsiella* and *Enterobacter* species.

Indicator antibiotic	*Klebsiella* species		*Enterobacter* species	
	
	Healthy dogs (24)	Diarrheic dogs (9)	Healthy dogs (24)	Diarrheic dogs (5)
Cefotaxime	12 (50%)	2 (22.2%)	4 (16.6%)	3 (60%)
Ceftriaxone	8 (33.3%)	4 (44.4%)	3 (12.5%)	2 (40%)
Ceftazidime	7 (29.1%)	4 (44.4%)	3 (12.5%)	1 (20%)
Aztreonam	2 (8.3%)	1 (11.1%)	-	-

### Confirmation of ESBL production

Among *Klebsiella* isolates, ESBL production was confirmed in 7 (21.2%) isolates. All these seven isolates were found resistant to at least one of the indicator cephalosporin used in the screening test but were found susceptible to combination of indicator cephalosporin with clavulanic acid or sulbactam in the confirmatory test. As clavulanic acid or sulbactam is beta-lactamase inhibitors, we can conclude that, in these seven isolates, the cephalosporin resistance mechanism could be mediated by beta-lactamase production. Clavulanic acid synergy (5 mm principle) was not detected in the remaining isolates, which might be due to the concurrent production of other non-ESBL beta-lactamases that confer resistance to beta-lactamase inhibitors [9,18]. The observed levels of ESBL phenotype among canine microbiota appeared to range from 1.4% to 90% in different studies, i.e., 1.4% in UK [19], 4% in Pennsylvania [18], 20% in Chili [20], 41.3% in China [21], and 90% in Australia [17]. ESBL phenotype was not detected in none of the *Enterobacter* isolates recovered in this study. In contrary, ESBL phenotype was reported in 9 out of 10 *Enterobacter* isolates recovered from opportunistic infections of dogs in Australia [17]. Difference in observed levels of beta-lactamase resistance and ESBL phenotype across various studies might be due to the variations in the methodology adopted, study population, extent of usage of third-generation cephalosporins, and drug pressure in the community.

### Detection of beta-lactamase (*bla*) genes

Detection of resistance genes using nucleic acid-based techniques has been of great use and has been shown to complement phenotypic results [11]. The overall incidence of beta-lactamase genes in *Klebsiella* and *Enterobacter* species was found to be 90.9% (30/33) and 72.4% (21/29), respectively. Incidence of beta-lactamase genes in canine microbiota was found to range from 17.5% to 82.2% across various studies, i.e., 17.5% in Tunisia [22], 56.5% in UK [19], and 82.2% in the Netherlands [23]. Frequency of beta-lactamase genes detected in *Klebsiella* and *Enterobacter* isolates recovered in the present study was given in Table 3. Among the *Klebsiella* isolates recovered from healthy dogs, one or more beta-lactamase genes were detected in a total of 21 (87.5%) isolates, whereas 3 (12.5%) isolates were found negative for all the genes. Among the *Klebsiella* isolates recovered from diarrheic dogs, all the isolates carried one or more of the beta-lactamase genes screened for. Overall, the incidence of *bla*_SHV_, *bla*_TEM_, *bla*_CTX-M_ Group 1, and *bla*_OXA_ genes was found to be 84.8%, 33.3%, 15.1%, and 6.1%, respectively, in *Klebsiella* species. In a study from France, *bla*_TEM_, *bla*_OXA_, and *bla*_CTX-M_ genes were reported to be detected in all the *Klebsiella* isolates recovered from the dogs [16].

**Table-3:** Frequency of beta-lactamase genes detected in *Klebsiella* and *Enterobacter* species.

Beta-lactamase gene	*Klebsiella* species		*Enterobacter* species	
	
	Healthy dogs (24)	Diarrheic dogs (9)	Healthy dogs (24)	Diarrheic dogs (5)
*bla_TEM_*	10 (41.6%)	1 (11.1%)	6 (25%)	1 (20%)
*bla_SHV_*	20 (83.3%)	8 (88.8%)	9 (37.5%)	5 (100%)
*bla_OXA_*	2 (8.3%)	-	2 (8.3%)	1 (20%)
*bla_AmpC_*	-	-	2 (8.3%)	2 (40%)
*bla_CTX-M_* Group 1	3 (12.5%)	2 (22.2%)	-	-

*bla_CTX-M_* Group-2, *bla_ACC_*, and *bla_MOX_* genes were not detected in the present study

Among the 24 *Enterobacter* isolates recovered from healthy dogs, one or more beta-lactamase genes were detected in a total of 16 (66.6%) isolates, whereas 8 (33.3%) isolates were found negative for all the genes. Among the 5 *Enterobacter* isolates recovered from diarrheic dogs, all the isolates carried one or more of the beta-lactamase genes screened for. Overall, the incidence of *bla*_SHV_, *bla*_TEM_, *bla*_AmpC_, and *bla*_OXA_ genes was found to be 48.2%, 24.1%, 13.7%, and 10.3%, respectively, in *Enterobacter* species. In a study conducted in Australia, *bla*_TEM,_ *bla*_SHV_, and *bla*_OXA_ genes were reported to be detected in 100%, 90%, and 10% of the *Enterobacter* isolates recovered from dogs, respectively [17]. In another study from Lisboa, 5.5% incidence of *bla*_AmpC_ gene was reported in *Enterobacter* species [24]. The phenotypic resistance to third-generation cephalosporins and monobactams detected in this study supported by its molecular confirmation by the detection of beta-lactamase genes probably indicates the need for careful selection and judicious usage of beta-lactam antibiotics in the treatment of canine infections in this region. The incidence rates are likely to increase further if strict control measures on the usage of third-generation cephalosporins were not implemented.

## Conclusion

This study reporting the beta-lactamase resistance among *Klebsiella* and *Enterobacter* species with due emphasis as indicators of antimicrobial resistance adds to the alarming beta-lactamase resistance reports among *Enterobacteriaceae* members worldwide. The alarming incidence of beta-lactamase resistance detected in this study might probably be the result of indiscriminate usage of third-generation cephalosporins in canine practice, which reflects the possible risk of therapeutic failures that may occur in the treatment of infections caused by *Klebsiella* and *Enterobacter* species.

## Authors’ Contributions

NMS is the student worked for M.V.Sc. thesis. BS as major guide and RKC as minor guide designed and supervised the research work. Manuscript was drafted and revised by NMS under the guidance of BS, RKC, and DS. All authors read and approved the final manuscript.
